# North Atlantic Oscillation in winter is largely insensitive to autumn Barents-Kara sea ice variability

**DOI:** 10.1126/sciadv.abg4893

**Published:** 2021-07-30

**Authors:** Peter Yu Feng Siew, Camille Li, Mingfang Ting, Stefan P. Sobolowski, Yutian Wu, Xiaodan Chen

**Affiliations:** 1 Geophysical Institute, University of Bergen, Bergen, Norway.; 2 Bjerknes Centre for Climate Research, Bergen, Norway.; 3 Lamont-Doherty Earth Observatory, Columbia University, Palisades, NY 10964, USA.; 4 NORCE Norwegian Research Centre, Bergen, Norway.; 5 Department of Atmospheric and Oceanic Sciences, Institute of Atmospheric Sciences, Fudan University, Shanghai, China.

## Abstract

Arctic sea ice extent in autumn is significantly correlated with the winter North Atlantic Oscillation (NAO) in the satellite era. However, questions about the robustness and reproducibility of the relationship persist. Here, we show that climate models are able to simulate periods of strong ice-NAO correlation, albeit rarely. Furthermore, we show that the winter circulation signals during these periods are consistent with observations and not driven by sea ice. We do so by interrogating a multimodel ensemble for the specific time scale of interest, thereby illuminating the dynamics that produce large spread in the ice-NAO relationship. Our results support the importance of internal variability over sea ice but go further in showing that the mechanism behind strong ice-NAO correlations, when they occur, is similar in longer observational records and models. Rather than sea ice, circulation anomalies over the Urals emerge as a decisive precursor to the winter NAO signal.

## Teaser

Accounting for internal variability reveals insights into mechanisms underlying an Arctic–mid-latitude teleconnection riddle.

## INTRODUCTION

**Fig. 1:**
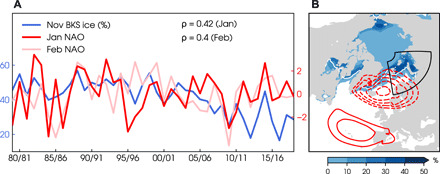
Relationship between Arctic sea ice in autumn and the winter NAO. (**A**) Time series of area-averaged November Barents-Kara sea ice concentration in % (blue) and the NAO index for January (red) and February (pink) from 1979/80 to 2018/19 from ERA5 reanalysis. Correlations (ρ) are calculated with linear trends removed. (**B**) Climatological sea ice extent (white contours showing 15% concentration) and interannual SD of the sea ice concentration in November (blue shading). The black box indicates the Barents-Kara Sea region (65°N to 85°N, 10°E to 100°E) for computing the sea ice index in (A). Regressions of January sea level pressure on the NAO index (red contours at 0.5-hPa intervals > |2| hPa, dashed contours indicate negative anomalies).

Autumn Arctic sea ice loss, especially over the Barents-Kara Sea, is associated with a negative North Atlantic Oscillation (NAO) the following winter (Fig. 1). This observed statistical relationship is a potential source of seasonal climate predictability for the North Atlantic region (*1*–*4*) and could lead to improved predictions of severe weather events, such as flooding and blizzards across Europe. Many different physical mechanisms have been proposed to explain the ice-NAO relationship and other related Arctic–mid-latitude teleconnections (*5*–*7*). Common among these mechanisms, however, are important roles for turbulent heat fluxes associated with sea ice loss, atmospheric blocking patterns over the Urals, and a weakening of the stratospheric polar vortex, as identified in both observational and modeling studies (*8*–*15*).

The ice-NAO relationship, simulated by climate models from the Coupled Model Intercomparison Project Phase 5 (CMIP5), differs substantially from that observed over recent decades, as reported in a number of previous studies. Long, preindustrial control simulations show very weak correlations between autumn/winter sea ice and the NAO (or Arctic Oscillation) compared to observations (*16*), with some indications that the so-called high-top models (those including a well-resolved stratosphere) produce slightly stronger signals (*17*). Historical simulations do show lagged relationships between the Barents-Kara sea ice reduction and a negative NAO, but the timing varies across models and is generally inconsistent with observations (*18*). While these studies indicate that ice-NAO linkages exist in some form in coupled models, these linkages seem not to set the long-term trends in European climate under future warming scenarios (*19*). The simulated ice-NAO relationship may be unrealistically weak because of model deficiencies, especially those related to near-surface heat fluxes in the stable boundary layers that are found at high latitudes during winter (*20*–*22*). However, another reason stems from internal variability, which has emerged as an important player in modulating atmospheric responses to sea ice variability.

Internal variability is the unforced and chaotic variability intrinsic to the climate system. On the time scales of interest here, most internal variability arises from atmospheric processes, such as the wave-mean flow interactions (*23*) that dominate the North Atlantic sector (*24*). These processes are suggested to cause intermittency in the ice-NAO relationship, which is detectable but not strong over the satellite era of observations from 1979 onward (*25*). Moreover, the relationship has been shown to be nonstationary in longer observational datasets going back to the 19th century, even switching signs during some periods (*26*). Modeling experiments that isolate the atmospheric response to sea ice perturbations report similarly equivocal results. Many perturbation experiments support a negative NAO response to sea ice loss mainly in the Barents-Kara Sea (*8*, *14*, *27*–*36*), but some show positive or neutral NAO responses (*37*–*39*). Regardless, the responses are usually detectable only when using very large sample sizes [many years or ensemble members; (*40*, *41*)], as would be expected in regions such as the North Atlantic where internal variability dominates. Furthermore, modeling results cannot explain the 2 to 3 months of delay from autumn ice anomalies to the winter NAO signal, indicating instead more immediate atmospheric responses (*42*, *43*). Together, these findings cast doubt on whether the ice-NAO relationship stems primarily from a forced response to sea ice variability.

If the ice-NAO statistical relationship is modulated by internal variability, as suggested by observational and modeling studies, then assessing whether it is well represented by coupled climate models requires some care. Internal variability in model simulations is not synchronized with reality and so can produce differences in the lag and sign of ice-NAO correlations compared to observations. When using long simulation periods, we average over various phases of internal variability, resulting in a more stable ice-NAO relationship. This can differ substantially from the same relationship derived from shorter observational records or simulations. Therefore, a direct comparison between the short 40-year satellite era and any given model simulation is not likely to be meaningful unless we consider how internal variability affects the answer.

In this study, we reevaluate how coupled climate models represent the lagged ice-NAO relationship from late autumn to winter, accounting for the role of internal variability. The main datasets used are preindustrial control simulations from CMIP5 and CMIP6, which are long enough to sample a large range of internal (unforced) climate variability, along with ensembles of shorter transient simulations to examine the influence of transient climate forcing. This analysis allows for a quantitative assessment of whether climate models capture the observed ice-NAO relationship and leads to new insights into the underlying physical mechanisms.

## RESULTS

### Variable ice-NAO correlations in climate models

**Fig. 2:**
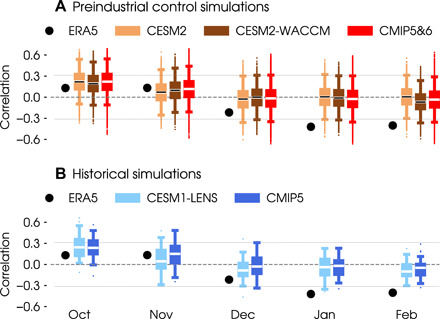
NAO correlations with autumn sea ice over the extended winter season. Lagged correlations between November Barents-Kara sea ice index (sign reversed such that positive values correspond to sea ice reduction) and the NAO from October to February. Black dots show correlations in ERA5 for the 40-year period from 1979 to 2019. Horizontal lines show the significance threshold at a 5% level using a two-tailed *t* test for a sample size of 40. (**A**) Preindustrial control simulations. Black horizontal bars show the correlations over the entire simulation from the Community Earth System Model version 2 (CESM2) (1200-year) and CESM2–Whole-Atmosphere Community Climate Model (WACCM) (499-year) models. Boxplots show the correlations from 10,000 bootstrap samples of 40 simulation years each, with CESM2 in orange, CESM2-WACCM in brown, and other CMIP5 and CMIP6 preindustrial control simulations (see Table 1) in red. The boxes indicate the 25th and 75th percentiles, whiskers indicate the 1st and 99th percentiles, and dots indicate outliers. (**B**) Historical simulations. Boxplots show the correlations in transient experiments for the 40-year period from 1979 to 2019, with the 40-member CESM1-LENS in light blue and other CMIP5 models (see Table 1) in dark blue.

The statistical relationship between autumn sea ice and the late-winter NAO is, at first glance, very weak in coupled climate models compared to reality. Figure 2A shows lagged correlations between the November sea ice and the NAO from October to February in the ECMWF Reanalysis version 5 (ERA5) reanalysis (which we will refer to as satellite-era observations) and in preindustrial control simulations from CMIP models (Table 1). The sign of the correlation coefficients is flipped to highlight the NAO phase associated with the anomalously low November sea ice. In the 40-year satellite record (black dots), low November sea ice is significantly correlated with negative NAO conditions in January and February. The climate models, however, show very weak NAO correlations in winter when calculated from the long control simulations, which range in length from 451 to 2000 years. For example, the January and February correlations are near zero (black horizontal bars) in simulations from the National Center for Atmospheric Research (NCAR) model, regardless of whether it is in a low-top [standard resolution in the stratosphere, Community Earth System Model version 2 (CESM2)] or high-top [well-resolved stratosphere, CESM2–Whole Atmosphere Community Climate Model (WACCM)] configuration. Other CMIP5 and CMIP6 preindustrial control simulations give consistent results (fig. S1). However, the observations and preindustrial control simulations have very different lengths, which influences the modulating effect of internal variability on the ice-NAO relationship.

**Table 1:** Models and experiments.

**Project**	**Experiment**	**Models**
CMIP6	Preindustrial control (length in years)	ACCESS-CM2 (500), ACCESS-ESM1-5 (900), BCC-ESM1 (451), CAMS-CSM1-0 (500), CESM2 (1200), CESM2-WACCM (499), CNRM-ESM2-1 (500), CanESM5 (1000), EC-Earth3-Veg (500), FGOALS-g3 (700), INM-CM5-0 (1201), IPSL-CM6A-LR (2000), MIROC6 (800), MPI-ESM1-2-HR (500), MPI-ESM1-2-LR (1000), MRI-ESM2-0 (701), NorESM2-LM (501), NorESM2-MM (500)
CMIP5	Preindustrial control (length in years)	CCSM4 (1200), CESM1-WACCM (400)
	Historical and RCP8.5 (1979–2019) single-member	ACCESS1.0, ACCESS1.3, CMCC-CESM, CMCC-CM, CMCC-CMS, CNRM-CM5, CSIRO-Mk3.6.0, CanESM2, FGOALS-s2, FIO-ESM, GISS-E2-H, GISS-E2-R, INM-CM4, IPSL-CM5A-LR, IPSL-CM5A-MR, IPSL-CM5B-LR, MIROC-ESM, MIROC-ESM-CHEM, MPI-ESM-LR, MPI-ESM-MR, MRI-CGCM3, NorESM1-M, NorESM1-ME, BCC-CSM1.1, MIROC5, GISS-E2-H-CC, GISS-E2-R-CC, GFDL-CM3, GFDL-ESM2G, GFDL-ESM2M, CCSM4, BNU-ESM, FGOALS-g2, HadGEM2-ES
	Historical and RCP8.5 (1979–2019) 40-member	CESM1-LENS

To put the comparison on a more equal footing, we use a bootstrapping test in which we randomly sample 40 years from a preindustrial control simulation (such that each sample is equivalent in length to the satellite-era observational period) and repeat this 10,000 times (see Materials and Methods). The distribution of correlations from the 10,000 bootstrap samples may then be compared to observations directly.

The bootstrapped ice-NAO correlations show a large spread encompassing both negative and positive values throughout the cold season (boxplots in Fig. 2A). Focusing first on the CESM2 (orange) and CESM2-WACCM (brown) models, the late-winter correlations are generally weak, with interquartile ranges that straddle zero and medians (white horizontal lines) that are close to zero, consistent with correlations calculated from the entire simulation period (black horizontal bars). Even with this considerable spread, there are very few 40-year samples that reproduce the observed ice-NAO relationship (black dots in Fig. 2A). In January and February, the observed correlations sit at the extreme end (lowest 1%) of the bootstrapped distributions, beyond the whiskers of the boxplots. Other CMIP5 and CMIP6 preindustrial control simulations show very similar spreads in bootstrapped ice-NAO correlations, resulting in the red boxplot (see fig. S1 for individual models). This aggregated CMIP spread is no larger than the spread of individual models, meaning that model diversity does not increase the uncertainty beyond that due to internal variability.

Extending the analysis from preindustrial control simulations to transient simulations, we find the same influence of internal variability on the ice-NAO relationship. Transient simulations apply a time-varying set of external forcings (anthropogenic greenhouse gases, aerosols, and insolation) that mimic reality in the period of interest. To account for internal variability, one can run a transient simulation multiple times with slightly different initial conditions to produce a multimember ensemble. One such “large ensemble” is the 40-member CESM1–Large Ensemble (LENS) [see Materials and Methods; (*44*)]. Figure 2B shows the ice-NAO correlations from CESM1-LENS for the period 1979–2019, with the ensemble median (white horizontal lines) and spread (light-blue boxplots) indicated. The NAO correlations in late winter are generally weak and exhibit a large spread comparable to the bootstrapped ice-NAO correlations from the preindustrial control simulations (Fig. 2A). Similar correlations are obtained using single-member transient simulations from other climate models (dark-blue boxplots in Fig. 2B), suggesting that internal variability is a more important factor than model differences for creating the CMIP spread. Together, these results show that external forcing does not appear to alter the effect of internal variability on the simulated ice-NAO relationship.

The results to this point indicate that internal variability strongly modulates the ice-NAO relationship on time scales of the satellite-era observational record, regardless of the presence of transient external forcing. Within the large range of possible ice-NAO behavior, the ice-NAO correlation over the past 40 years is a rare occurrence. It is also unusual in the context of observational products covering the 20th century (*26*). Thus, the observed autumn ice–winter NAO relationship in recent decades is at the edge of the variability represented by long climate model simulations and long observational records (fig. S1) (*26*).

### Weak linkages through atmospheric circulation, none apparent through sea ice

The physical mechanisms invoked to explain the teleconnection pathway from autumn ice to winter NAO can guide further explorations of the large spread in model behavior. All pathways begin with sea ice reductions, causing increased turbulent surface fluxes as the ocean gives up more heat to the atmosphere (*45*). This heating perturbation is thought to lead to various atmospheric circulation changes, depending on the pathway, including atmospheric blocking (a quasi-stationary high pressure weather pattern) over the Urals, increased upward wave activity flux, and a weakening of the stratospheric polar vortex (*14*, *29*, *30*). However, these causal linkages are controversial even for the satellite era (*25*, *39*, *42*). We examine circulation features in the simulations by partitioning the 40-year bootstrap samples according to hypothesized linkage mechanisms. This helps to infer the underlying dynamics responsible for generating the large model spread in ice-NAO correlations.

**Fig. 3:**
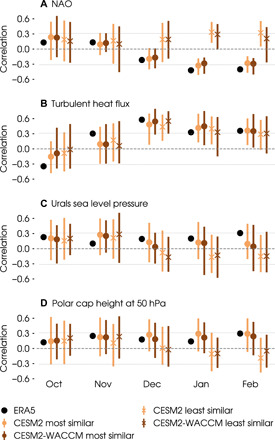
Atmospheric circulation anomalies in bootstrap samples partitioned by ice-NAO behavior. Lagged correlations between November Barents-Kara sea ice index (sign reversed such that positive values correspond to sea ice reduction) and indices of (**A**) NAO, (**B**) turbulent surface heat flux (positive from ocean to atmosphere), (**C**) Urals sea level pressure, and (**D**) polar cap height at 50 hPa, all from October to February. Black dots show correlations in ERA5 for the 40-year period from 1979 to 2019. Vertical bars show the range and markers show the median of correlations in the 100 bootstrap samples with an ice-NAO relationship most (circles) and least (crosses) similar to the ERA5 reanalysis from the CESM2 (orange) and CESM2-WACCM (brown) preindustrial control simulations. Horizontal lines show the significance threshold at a 5% level using a two-tailed *t* test for a sample size of 40. Other CMIP5 and CMIP6 preindustrial control simulations give consistent results (see fig. S2).

The observations provide only weak statistical evidence of a teleconnection via the so-called stratospheric pathway from the ice to the NAO. The clearest signal is the first step of the linkage: Low November ice is associated with positive (ocean to atmosphere) turbulent heat fluxes through the winter in the satellite era (black dots in Fig. 3B). Later in the winter, the associated circulation features are of the expected sign: positive Urals sea level pressure (i.e., strengthening of blocking over the Urals) and positive 50-hPa polar cap height (i.e., weakening of the polar vortex) from December to February (Fig. 3, C and D). However, the correlations are not statistically significant in January and only nearly significant in February, which is consistent with conclusions from casual discovery analyses that the pathway is detectable but intermittent in the satellite period (*25*). Note, in addition, that low November ice is preceded by negative anomalies in turbulent heat flux and positive anomalies in the NAO and Urals sea level pressure, consistent with the fact that sea ice responds to atmospheric variability (*47*–*49*).

The coupled models also show some indication of teleconnection pathways through atmospheric circulation changes but with considerable uncertainty due to internal variability. Because the models exhibit such varied ice-NAO behavior, we first categorize the bootstrap samples from the preindustrial control runs on the basis of how closely their ice-NAO relationship resembles observations (measured by ice-NAO correlations over the November-February cold season; see Materials and Methods). In this way, we identify a group of samples that are most similar to the observations (circles in Fig. 3A for CESM2 and CESM2-WACCM; see fig. S2 for other CMIP models), and a group of samples that are least similar to the observations, i.e., showing positive ice-NAO correlations in late winter (crosses). The bootstrap samples in the “most similar” group tend to associate reduced autumn sea ice with higher Urals sea level pressure (Fig. 3C) and weakening of the polar vortex in winter (Fig. 3D), while those in the “least similar” group tend to show opposite signals. Looking across all CMIP models, there is also a slight but consistent tendency for the most similar group to exhibit higher Urals sea level pressure in November, with CESM2-WACCM being the only exception (fig. S2C), suggesting that the Urals blocking precedes a warmer polar vortex and negative NAO conditions in winter. Although the two groups show differences in signals from autumn to winter, the correlations between ice and these circulation features exhibit large within-group variability: The distributions of both groups straddle zero, and they overlap substantially. All these messages apply to variability across the longer 20th-century observational record, which behaves similarly to the CMIP simulations (fig. S2). In other words, the teleconnection mechanism is not clearly distinct, even at the two extremes of ice-NAO behavior.

**Fig. 4:**
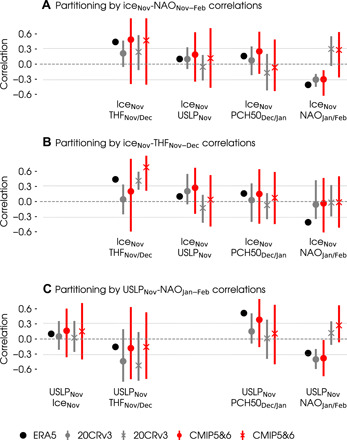
Mechanisms underlying the ice-NAO relationship. Lagged correlations between key indices in proposed pathways, including Barents-Kara sea ice (ice, sign reversed such that positive values correspond to sea ice reduction), turbulent surface heat flux (THF, positive from ocean to atmosphere), Urals sea level pressure (USLP) and polar cap height at 50 hPa (PCH50). Black dots show correlations in ERA5 for the 40-year period from 1979 to 2019. Vertical bars show the range and markers show the median of correlations in (**A**) 100 bootstrap samples with an ice-NAO relationship most (circles) and least (crosses) similar to the ERA5 reanalysis, (**B**) 100 bootstrap samples with the most (crosses) and least (circles) positive correlations between November ice and November to December turbulent surface heat flux, and (**C**) 100 bootstrap samples with the most negative (circles) and positive (crosses) correlations between November Urals sea level pressure and January to February NAO from the 20th Century Reanalysis (gray, 20CRv3), and CMIP5 and CMIP6 preindustrial control simulations (red, CMIP5&6). Horizontal lines show the significance threshold at a 5% level using a two-tailed *t* test for a sample size of 40.

However, the teleconnection from reduced ice to negative NAO, when it exists, does not seem to include a physical link through turbulent heat fluxes. Both most similar and least similar bootstrap groups exhibit positive correlations with turbulent heat fluxes from November to February (Fig. 3B; see Fig. 4A and fig. S2B for other preindustrial control simulations and the 20th-century reanalysis). This means that autumn sea ice reduction does induce heat fluxes from the ocean to the atmosphere, but the resulting NAO state in late winter can be negative (as in the most similar group and satellite-era observations) or positive (as in the least similar group). In other words, the sign of the late-winter NAO does not seem to be associated with autumn heat flux anomalies arising from sea ice variability. This result stems directly from the fact that surface heat fluxes may drive changes in sea ice and may be driven by sea ice changes, with the former dominating in both observations and models (*50*, *51*). Overall, this result is consistent with previous modeling experiments indicating a lack of strong, forced mid-latitude circulation responses to sea ice variability in the Arctic-Atlantic sector (*35*, *39*, *42*, *46*, *51*–*60*).

The message becomes even clearer if we partition the bootstrap samples according to turbulent heat flux instead of the late-winter NAO (Fig. 4B and fig. S3B). Doing so reveals that the low November sea ice can be associated with both positive and negative turbulent heat fluxes. Furthermore, it is the “less positive” group (purple in fig. S3 and circles in Fig. 4B) that tends to show stronger Urals blocking in November and December. Because this group represents bootstrap samples dominated by atmosphere-driven surface heat flux variability (reduced ice and less heat from ocean to atmosphere), the implication is that atmospheric variability is a common driver of autumn Urals blocking and autumn sea ice reduction, consistent with previous studies (*42*, *61*, *62*). No stratospheric or NAO signals in winter are apparent in this analysis, but this is because the flux partitioning still includes a link to November ice, which has now been shown to play a negligible role. Partitioning by correlations to Urals sea level pressure rather than autumn ice proves more illuminating and reveals that blocking in autumn is followed by the weakening of the polar vortex and a negative NAO in late winter (Fig. 4C). Together, these results point to Urals blocking as a robust precursor to wintertime polar vortex and NAO signals and demonstrate that this chain of circulation changes can account for the range of lagged ice-NAO correlations seen in models and longer observational records.

**Fig. 5:**
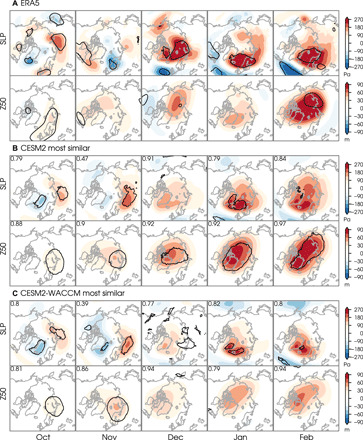
Atmospheric circulation patterns associated with ice-NAO teleconnection pathway suggested by observations. Lagged regressions (shading) of sea level pressure (SLP) and 50-hPa geopotential height (Z50) onto November Barents-Kara sea ice index (standardized and sign reversed such that positive values correspond to sea ice reduction) from October to February. (**A**) Regression maps from ERA5 reanalysis for the 40-year period from 1979 to 2019. Contours enclose significant values at the 5% level using a two-tailed *t* test. (**B** and **C**) Composite of regression maps from the 100 bootstrap samples with an ice-NAO relationship most similar to ERA5 reanalysis from the (B) CESM2 and (C) CESM2-WACCM preindustrial control simulations. Contours enclose regions where more than 30% of the bootstrap samples show significant values at the 5% level using a two-tailed *t* test. Numbers in the top left corners of the panels indicate the pattern correlations (poleward of 30°N) with ERA5.

Keeping focus on gaining mechanistic understanding, we examine the spatial signatures of the circulation anomalies that distinguish the simulated 40-year samples with the ice-NAO behavior most similar to observations. We perform lagged grid-point regressions of October-to-February sea level pressure and 50-hPa geopotential height onto the November sea ice index. Consistent with the analysis in Fig. 3, we see signatures of a high pressure extending across the Urals region and a weaker polar vortex throughout autumn and winter in both the CESM2 (Fig. 5B) and CESM2-WACCM (Fig. 5C), as in satellite-era observations (Fig. 5A). These circulation features are generally not robust, as indicated by the small regions exhibiting statistical significance, but show high-spatial pattern correlations with the observations (upper-left corners of individual panels). The polar vortex weakens more in the low-top CESM2 than the high-top CESM2-WACCM, which is the opposite of what was seen in sea ice removal experiments (*14*, *30*), across low/high-top CMIP models (*17*), including the previous generation of the NCAR model (low-top CCSM4 and high-top CESM1-WACCM in fig. S4). We note that the low-top CESM2 represents stratospheric variability quite well (*63*). Thus, a well-resolved or well-represented stratosphere does seem to help a model’s ability to capture the large-scale circulation signals associated with the polar vortex weakening.

**Fig. 6:**
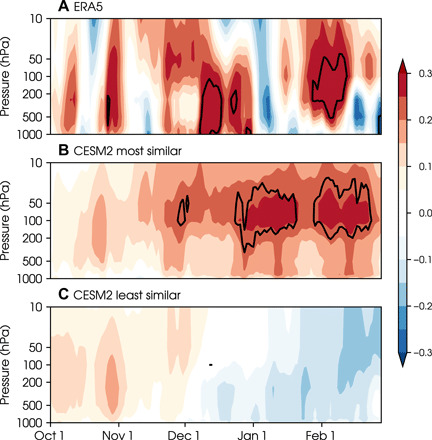
Downward propagation signals from stratosphere to troposphere. Lagged correlations (shading) between November Barents-Kara sea ice index (sign reversed such that positive values correspond to sea ice reduction) and polar cap height (poleward of 70°N) from October to February. (**A**) Correlation profiles in ERA5 reanalysis for the 40-year period from 1979 to 2019. Contours enclose significant values at the 5% level using a two-tailed *t* test. Composites of correlation profiles from the 100 bootstrap samples with an ice-NAO relationship (**B**) most and (**C**) least similar to ERA5 reanalysis from the CESM2 preindustrial control simulations. Contours enclose regions where more than 30% of the bootstrap samples show significant values at the 5% level using a two-tailed *t* test.

The fact that the polar vortex and sea level pressure patterns in Fig. 5 lack statistical robustness suggests that there is uncertainty in the downward propagation mechanism by which signals in the stratosphere are proposed to influence the troposphere (*64*), even in the most similar bootstrap samples. Lagged daily correlations between autumn sea ice variability and polar cap height anomalies in the satellite record (Fig. 6A) show low November sea ice associated with positive height anomalies in the stratosphere (indicating an anomalously weak polar vortex) that descend into the troposphere from mid-November through early January. This correlation structure does not represent specific downward propagation events because their exact timing during the winter season is highly unpredictable and thus vary from winter to winter. However, comparing the structure of the most similar to least similar bootstrap samples (Fig. 6, B and C, for CESM2 and fig. S5 for CESM2-WACCM and CESM1-WACCM), we see a composite signature of more downward propagation events in the most similar samples.

## DISCUSSION

This study reevaluates how climate models represent the observed ice-NAO relationship in the 40-year satellite-era record. The mechanistic approach adopted here helps to better understand why the relationship exists and what its main drivers are. Looking at simple lagged correlations, it is clear that the satellite record exhibits an unusually strong relationship between reduced ice in autumn and the negative NAO in winter, compared to climate models and in the context of the 20th century (*17*, *18*, *26*, *46*). By acknowledging the presence of internal variability, we can bootstrap long simulations or observational records to show that they exhibit comparably large spreads in ice-NAO correlations, with only a small number of bootstrap samples (<1%) resembling satellite-era observations. This is true regardless of stratospheric representation and in both control and forced transient simulations.

The fact that the bootstrap sampling distributions cover such a wide range of behavior makes them ideal test beds for investigating proposed mechanisms for ice-NAO linkages. The medians of the bootstrapped ice-NAO correlations in late winter sit around zero (Fig. 2), indicating no preferred sign for the atmospheric responses. This already points to an important role for internal variability in driving periods of strong ice-NAO correlations, whether positive or negative. Using December rather than November ice anomalies shifts the median correlations such that they are slightly negative but still near zero given the spread (fig. S6 for CMIP6) [(*17*) for CMIP5].

There might still exist a causal ice-to-NAO mechanism that operates intermittently to produce the strong negative NAO correlations in the bootstrap distribution (e.g., those capturing the satellite-era observed relationship), but we find no support for this. Causality would require a physical linkage from ice to surface turbulent heat flux anomalies (*50*, *51*); reduced autumn ice should be associated with stronger ocean-to-atmosphere fluxes, which then lead to Urals blocking and polar vortex weakening. However, partitioning the bootstrap distribution by ice-flux correlations shows no sign of this (Fig. 4B and fig. S3). It is the bootstrap samples in which reduced ice is associated with weaker (or negative) heat fluxes that exhibit a tendency for Urals blocking (Fig. 4B and fig. S3), that is, the samples in which atmospheric variability is a common driver for the autumn ice and heat flux anomalies. A final piece of evidence for ruling out the ice-driven teleconnection is by repeating the correlation analysis with scrambled sea ice and atmospheric indices representing the intermediate steps of the pathway (in other words, pairing sea ice and atmospheric variables from different bootstrap samples). The spread in scrambled correlations is nearly identical to the spread in the original correlations (e.g., for CESM2; compare fig. S7 and Fig. 2A), indicating that the relationships can arise entirely from internal variability.

The fact that climate models and longer observational records yield comparable spread in ice-NAO correlations on a 40-year time scale suggests that the strong negative correlation over the most recent 40-year period is indeed unusual. However, it is possible that sea ice can drive a winter NAO response through teleconnection pathways that global climate models fail to capture (*65*). There are well-known deficiencies in models that influence how surface (ice) perturbations are communicated to the atmosphere, including problems with Arctic boundary layers (*20*, *21*) and overly weak signal-to-noise ratio (*66*).

The question remains as to why the ice-NAO relationship is so strong in the satellite era. The Urals blocking precursor may provide a clue. Urals blocking has been suggested to drive polar vortex weakening and negative NAO conditions in observational and modeling studies (*42*, *61*). Recent decades may have seen Urals blocking promoting Barents-Kara sea ice loss in autumn but with confounding influences, such as ocean warming creating positive surface heat flux anomalies. This would result in an artificial ice-flux correlation in autumn (Fig. 4B), leading to an incorrect interpretation of causality for the winter NAO signal (Fig. 4C).

In summary, this study provides new insights into the mechanism behind the lagged ice-NAO relationship in models and observations. Climate models and long observational records show a large spread in ice-NAO correlations and occasionally exhibit periods of low autumn ice occurring with a negative winter NAO (such as the satellite-era observations). These periods are marked by circulation signals that are consistent with proposed teleconnection mechanisms, including atmospheric blocking over the Urals and a weakening of the polar vortex. However, the winter circulation changes are primarily driven by atmospheric variability rather than autumn sea ice variability, with Urals blocking emerging as a decisive precursor. Barnes and Screen (*67*) posed three questions to guide explorations of whether Arctic change influences mid-latitude weather: “Can it? Has it? Will it?”. Borrowing this framework for the ice-NAO relationship, we know that many idealized ice-perturbation modeling experiments answer yes to the first question—changes in sea ice can drive an NAO response. However, in the coupled system, variability arising from interactions within or between the atmosphere, ice and ocean appear to overwhelm any ice-driven NAO signal. Thus, the answer to “Has it?” is most likely no. While some studies suggest that autumn sea ice conditions are a potential predictor of the winter NAO in dynamical [e.g., (*1*)] and empirical models [e.g., (*3*)], it appears that the underlying relationship is nonstationary, which undermines its utility. As sea ice continues to decline into the future, the response may become stronger as some studies suggest (*22*, *28*). However, further investigation into alternative mechanisms and whether these change in a systematic manner in the future are likely needed to provide a comprehensive answer to “Will it?”.

## MATERIALS AND METHODS

### Observations and models

The observational basis for this study is the European Centre for Medium-Range Weather Forecasts (ECMWF) ERA5 reanalysis dataset (*68*) covering the period from 1979 to 2019, and the National Oceanic and Atmospheric Administration–University of Colorado Boulder’s Cooperative Institute for Research in Environmental Sciences–U.S. Department of Energy (NOAA-CIRES-DOE) 20th Century Reanalysis V3 (*69*, *70*) covering the period from 1900 to 2015. For climate modeling results, we used a range of preindustrial control simulations from the CMIP5 (*71*) and CMIP6 (*72*). The long preindustrial control simulations provide an opportunity to sample internal variability in an unforced climate state. To check the sensitivity of the results to transient external forcing from changes in greenhouse gases, land use, aerosols, and insolation, we also used transient simulations for the period 1979–2019 from CMIP5 (*71*), including the NCAR CESM large ensemble experiment (CESM1-LENS) (*44*). The transient simulations include the period 1979–2005 taken from the CMIP5 historical simulations and the period 2006–2019 taken from the CMIP5 Representative Concentration Pathway 8.5 (RCP 8.5) scenario simulations. The CESM1-LENS is a special transient experiment comprising 40 members run by the same model under the same external forcings but with slightly different initial conditions; thus, the members can be considered alternative realizations of the period of interest arising from internal climate variability. See Table 1 for details of all model simulations used.

### Data and statistical methods for characterizing ice-NAO relationship

To investigate the ice-NAO relationship in these datasets, we defined indices to represent variability in Arctic sea ice and the NAO. For Arctic sea ice, we focused on the Barents-Kara Sea, which is the region with the strongest sea ice variability in the autumn and winter seasons, in both observations and models (fig. S8). We removed the climatological monthly/daily means in all data from all reanalysis and simulations. We further detrended all data from the reanalysis and transient simulations. The results are not sensitive to the detrending method (i.e., removing the linear trend or 10-year running mean) or to whether decadal detrends in preindustrial simulations are removed or not. Here, we used the monthly sea ice concentration and sea level pressure to calculate the following:

1) Barents-Kara sea ice (ice): sea ice area fraction area-averaged over 65°N to 85°N, 10°E to 100°E (*31*);

2) NAO: difference in area-averaged sea level pressure between two regions, 55°N to 85°N, 40°W to 20°E and 25°N to 55°N, 50°W to 10°E (*3*), standardized by subtracting the mean and dividing by the SD. Similar results were obtained using more standard NAO indices [(i.e., station-based or Empirical Orthogonal Function (EOF)-based; see (*73*)].

We calculated lagged correlations between the Barents-Kara sea ice index in November (ice_Nov_) and the monthly NAO index for each month from October to February (NAO_Oct–Feb_) in the observations and the preindustrial control simulations. In the simulations, we first examined the ice_Nov_-NAO_Oct–Feb_ correlations over the entire simulation period to assess the long-term relationship. Then, we examined these correlations within 10,000 bootstrap samples, each comprising 40 winter seasons (October to February) randomly drawn from the entire simulation without replacement. The bootstrapping approach generates a large number (10,000) of ice_Nov_-NAO_Oct–Feb_ correlations based on periods that are equivalent in length to the observations, providing an estimate of the range of ice-NAO correlations due to internal climate variability. The random sampling assumes that every year is equivalent, which is reasonable for unforced preindustrial control simulations. Similar analyses were repeated for the consecutive 40-year periods, and the result was found to hold, meaning that it is not sensitive to the presence of lower-frequency internal variability.

Similar analyses were carried out with the CESM1-LENS and single-member CMIP5 transient simulations but without bootstrapping because the presence of external forcing requires us to use the specific 40-year period of interest (1979–2019). The CESM1-LENS experiment provides some measure of the range of internal variability over its 40 members.

### Data and statistical methods for investigating proposed ice-NAO mechanisms

Bootstrap samples were classified according to how well they reproduce the observed relationship between autumn sea ice and the late-winter NAO to investigate the proposed underlying mechanisms. Considering the monthly ice_Nov_-NAO_Nov–Feb_ correlations (i.e., NAO lags of 0 to +3 months relative to ice_Nov_), we calculated the root mean square error (RMSE) to quantify the difference between the ERA5 reanalysis (observations) and the models. Bootstrap samples with smaller RMSE values were determined to better resemble the observations. We repeated the procedure for each bootstrap sample to identify the 100 bootstrap samples (1%) with an ice-NAO relationship most and least similar to the observations. Similar results were obtained when defining groups according to ice_Nov_-NAO_Dec–Feb_ or ice_Nov_-NAO_Jan–Feb_ correlations and when using alternative methods for identifying the least similar (or, more accurately, “opposite to observations”) samples.

To investigate proposed mechanisms in these samples, we defined a selection of indices that represent the key features of the teleconnection pathways (*11*). We used monthly sea level pressure, monthly sensible and latent heat flux, and monthly and daily geopotential height to derive the following indices:

1) Turbulent heat flux: sum of surface sensible and latent heat flux area-averaged over 65°N to 85°N, 10°E to 100°E, with positive defined as heat flux from the ocean to the atmosphere;

2) Urals sea level pressure: sea level pressure area-averaged over 45°N to 70°N, 40°E to 85°E (similar results were found using geopotential height at 500 hPa);

3) Polar cap height at 50 hPa: monthly geopotential height at 50 hPa area-averaged from 65°N poleward;

4) Polar cap height in all levels: daily geopotential height from 10 to 1000 hPa, area-averaged from 70°N poleward at each level.

Several metrics were used to compare the indices from the selected bootstrap samples with those from the observations: lagged correlations with ice (monthly), gridpoint regressions onto ice (monthly) to investigate spatial signatures, and lagged height-time correlations between ice and polar cap height (daily) to investigate the downward influence of the stratosphere on the troposphere. To further investigate the underlying mechanisms, we partitioned the 40-year bootstrap samples according to hypothesized linkage mechanisms on the basis of correlations between November ice and November to December turbulent heat flux, and correlations between November Urals sea level pressure and January to February NAO.

## SUPPLEMENTARY MATERIALS

Supplementary material for this article is available at http://advances.sciencemag.org/cgi/content/full/7/31/eabg4893/DC1
